# Interaction of Sensitivity, Emotions, and Motivations During Visual Perception

**DOI:** 10.3390/s24227414

**Published:** 2024-11-20

**Authors:** Sergey Lytaev

**Affiliations:** Department of Normal Physiology, St. Petersburg State Pediatric Medical University, 194100 Saint Petersburg, Russia; physiology@gpmu.org; Tel.: +7-812-416-5226

**Keywords:** visual evoked potentials, change in sensitivity, emotions, reversible chessboard pattern, personality testing, augmentation, reduction

## Abstract

When an organism is exposed to environmental stimuli of varying intensity, the adaptive changes in the CNS can be explained by several conceptual provisions: the law of motivation–activation by Yerkes and Dodson, the laws of force and pessimal protective inhibition, and the theory of emotion activation. Later, reinforcement sensitivity theory was developed in the fields of psychology and psychophysics. At the same time, cortical prepulse inhibition (PPI), the prepulse inhibition of perceived stimulus intensity (PPIPSI), and the augmentation/reduction phenomenon were proposed in sensory neurophysiology, which expanded our understanding of consciousness. The aim of this study was to identify markers of levels of activity of cognitive processes under normal and in psychopathological conditions while amplifying the information stimulus. For this purpose, we changed the contrast level of reversible checkerboard patterns and mapped the visual evoked potentials (VEPs) in 19 monopolar channels among 52 healthy subjects and 39 patients with a mental illness without an active productive pathology. Their cognitive functions were assessed by presenting visual tests to assess invariant pattern recognition, short-term visual memory, and Gestalt perception. The personalities of the healthy subjects were assessed according to Cattell’s 16-factor questionnaire, linking the data from neurophysiological and cognitive studies to factors Q4 (relaxation/tension) and C (emotional stability). According to the N_70_ and N_150_ VEP waves, the healthy subjects and the patients were divided into two groups. In some, there was a direct relationship between VEP amplitude and contrast intensity (21 patients and 29 healthy persons), while in the others, there was an inverse relationship, with a reduction in VEP amplitude (18 patients and 23 healthy persons). The relationship and mechanisms of subjects’ cognitive abilities and personality traits are discussed based on the data acquired from the responses to information stimuli of varied intensity.

## 1. Introduction

From birth, many stimuli and environmental conditions impact an organism, causing responses and adaptive changes in its physiology, including the central nervous and sensory systems. The new connections and nerve centers that are formed affect executive functions, including perception, attention, memory, consciousness, and others. Since the beginning of the 20th century, in CNS and psyche research, many ideas about adaptive reactions to increasingly intense external stimuli have been formed. One of the first theories proposed was the motivation–activation theory described by Yerkes and Dodson. At a particular moment, optimal motivation is associated with the labor required to achieve the maximum effect; thus, performance effectiveness is dependent on the level of motivation [1,2,3,4]. Next, the laws of force and then pessimal protective inhibition were formed [4]. One of the leading mechanisms is the activation of inhibitory mechanisms regulating reflex activity in psychoneurological patients in order to preserve internal energy resources [5,6,7,8,9]. The laws of force and pessimal protective inhibition represent universal mechanisms explaining adaptive dynamic shifts [5]. To a lesser extent, pessimal inhibition, which ensures compensatory restructuring in psychopathology, is associated with the regulation of executive cognitive functions when the body is exposed to extreme stimuli [10].

One of the most famous psychological ideas in the field of personality behavior is reinforcement sensitivity theory (RST). According to RST, the basis of reactions to motivational and currently important stimuli [5,11,12] is the interaction of the defense behavior systems. These include fight–flight–freeze (FFFS), behavioral inhibition (BIS), and the behavioral approach system of positive emotions (BAS). In addition, according to RST, individual variations in the strength of responses and/or changes in the sensitivity of all these systems are associated with basic personality differences [13]. On this basis, personality scales were formed to assess temporarily stable tendencies during the activation of theBIS, BAS, and FFFS [13,14].

The behavioral approach system regulates the perception of positive/useful stimuli and provides enthusiasm, interest, and joy. The BAS is divided into a number of processes. Thus, interest in rewards and persistence in achieving a goal are early processes, and reactivity to rewards and impulsivity are considered to be late behavioral processes that act as predictors of attentional activation in the perception of negative images. The early processes do not have such a connection [5,12,15,16].

The fight–flight–freeze system [13,14] regulates negative behavioral effects, such as punishment/aversive stimuli accompanied by fear. The BIS balances motivational conflicts and is supported by anxious feelings. In particular, the perception of an animal (e.g., a dog) can be accompanied by varied motivations, emotions, and behavioral reactions in different people. Some subjects (e.g., children) have positive emotions and want to approach the animal and even touch it. In other cases, fear and avoidance reactions may appear. In addition, anxiety and surprise regarding the safety of approaching the animal may develop [13,14,17].

Subjects with a strong behavioral approach system have a low threshold for perceiving positive emotions, while those with a strong behavioral inhibition system have a low threshold for perceiving anger, fear, and disgust, which is also associated with the power of the EEG alpha rhythm. Additionally, subjects with a strong BAS have increased sensitivity to perceiving negative images. In a number of studies, data were assessed based on the power of the EEG alpha rhythm and the amplitude of event-related potentials (ERPs) [18,19,20,21].

Traditionally, in such studies, the amplitude–temporal characteristics of the visual evoked potential are assessed by the perception of the physical characteristics of the stimulus. These are waves with a latent period of 100–150 ms (N_100_ or N_150_) and a maximum negative peak between 200 and 300 ms (N_200_) before the formation of cognitive waves with a peak latency of more than 300 ms. In particular, the attention mechanisms of the BAS and FFFS were studied during image perception with subsequent one-way repeated ANOVA. When assessing the average amplitudes of N_100_ and N_200_, it was found that higher VEP amplitudes reflect the activation of attention to a specific category of images for subjects with different BASs and FFFSs [20].

To identify the changes in sensitivity to sensory processes, some researchers assess the influence of preimpulsation on perception and attention mechanisms, with subsequent increases in stimulus power [22,23,24]. Such studies are carried out in the clinic, but more often in psychiatric facilities [25], and the findings are applied to areas such as sports [26].

It is known that a weak stimulus presented immediately before a more intense one reduces the amplitude of the cortical N1–P2 wave, as well as the perceived intensity of the new, stronger stimulus. Such effects are called cortical prepulse inhibition (PPI) and prepulse inhibition of perceived stimulus intensity (PPIPSI), respectively. Both of these phenomena are used to study sensory gating in clinical and non-clinical populations. It has been concluded that PPIPSI is partly related to the allocation of attentional resources in monitoring cortical channels that process stimulus intensity features, such as the N1–P2 complex of auditory EPs [27].

Prepulse inhibition (PPI) is a reduction in the acoustic startle reflex (ASR) when a fearful stimulus is preceded by a weaker, non-fearful stimulus (i.e., prepulse). Research has shown that PPI may have a corticofugal origin due to the selective attention given to fear-induced prepulses. The results suggest that PPI is influenced by emotional and perceptual spatial attention and by gender. This highlights the additional application of PPI to measure top-down attentional regulation, which may improve the diagnosis of affective disorders, such as anxiety, depression, and post-traumatic stress disorder [28].

Other studies have addressed this issue by investigating whole-brain oscillatory activity using simultaneous functional magnetic resonance imaging, electroencephalography [29], and magnetoencephalography techniques [30].

Thus, various behavioral states cannot be analyzed only by using psychological testing and calculating personality scales. Behavioral scale scores largely reflect characteristics that are relatively stable over time. In this case, the online assessment of the fast activation of the BIS and the BAS uses various neurophysiological techniques, among which the amplitude–time characteristics of EEG and EP wave processes are considered optimal [3,20,31,32,33].

In addition, in its genesis and external manifestation, reinforcement sensitivity theory correlates with the kinesthetic phenomenon from sensory physiology and psychology “Augmentation/Reduction” (A/R) [4,14,31,34]. According to the provisions of this phenomenon, in some cases, subjects exaggerate the real weight of an object, and in others, they underestimate the weight. The former were accordingly called “augmentors”, and the latter were called “reducers” [3,12,31,35].

This research assessed the productivity of executive cognitive functions via specific sensory processes by measuring the responses to stimulation from reversible chessboard patterns of varying intensity and visual evoked potentials and by subsequently assessing emotions, motivations, and personal characteristics.

## 2. Materials and Methods

This study involved healthy subjects aged 20–22 years in the control group and 39 patients with a psychopathology with varying clinical presentations placed in the research group (mean age: 38.5; range: 21–53). The diagnosis of schizophrenia was verified during a psychiatric examination in accordance with ICD-11 (06. Mental and behavioral disorders. Schizophrenia or other primary psychotic disorders) in a psychiatric institution (hospital), where the patients were observed. The average medical history spanned 9.8 ± 3.1 years. For professional selection (the healthy subjects) and for routine medical and social examination (the patients), they underwent a routine psychiatric examination at an outpatient psychoneurological dispensary. During an examination, pharmacological support for the patients was reduced under the supervision of psychiatrists.

The VEPs were recorded in 19 monopolar channels according to the international 10/20 system using a neuromapper with reference electrodes on the earlobes. The electric dipole represented the potential difference between sites on the scalp and the reference electrodes. The signals were analyzed at a sampling frequency of 500 Hz and a bioamplifier bandwidth from 0.5 to 50 Hz, which are traditionally used for recording long-latency evoked potentials. The quality of the recorded VEPs was identified using automatic indicators. After this, the biosignals with background EEG were recorded for verification. Then, the bioamplifier switched to recording super-averaged evoked potentials. Reversible chessboard patterns with the subjects’ eyes open were presented on the display screen with a stimulation frequency of 1 Hz. Three series of patterns were used, differing in the contrast power of the chessboard elements: low, medium, and high [4].

The spatiotemporal characteristics of the evoked potentials were estimated at the analysis epoch of 400.0 ms from the stimulus presentation using topographic brain mapping data. The amplitude and peak latency (PL) of the N_70_ and N_150_ components were measured by hovering the cursor over the peaks of the averaged VEPs. The distance from the isoline to the peak was digitized. The most informative indicators in the study groups were calculated using stepwise discriminant analysis. Significant parameters were selected based on the F-statistics criterion of one-way ANOVA (F > 4.0). The stability of the spatiotemporal characteristics of the assessed VEP components was calculated using factor analysis. Parameters with a low factor ordinal number in relation to the proportion of reproducible variance were considered to be maximally stable.

A study of several cognitive executive functions associated with the recognition of visual images of different modalities was conducted by presenting fragments of familiar images on a display screen under conditions that hindered perception. To test short-term visual memory, independent of language abilities, 32 complex geometric figures designed by Perret [36] were used, which form a complete image that has no name. After giving appropriate instructions, the fragments were presented to the subjects one after another with an interval and exposure time of 500.0 ms. Subjects then attempted to identify the figure. In addition, when a time deficit was applied (exposure from 4.0 ms to 3.0 s), images with an incomplete set of features were presented [4,37].

The emotional sphere was assessed using the 16-factor questionnaire of the Cattell test. Subsequently, analysis and correlation with the neurophysiological data were performed on two factors: C (emotional stability) and Q4 (relaxation and tension).

**Figure 1:**
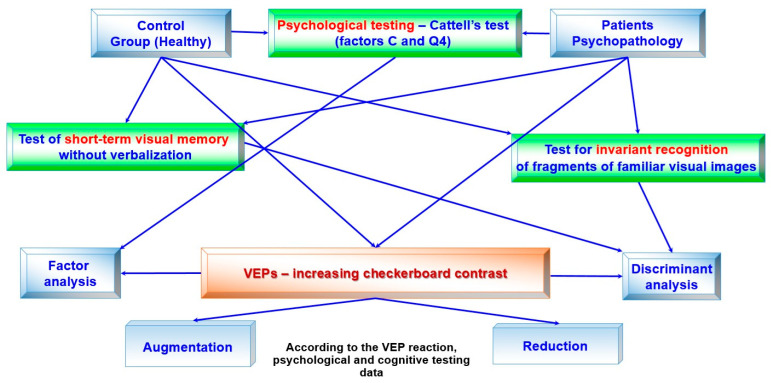
Block diagram of the research, processing of results.

A general block diagram of the conducted study is presented in Figure 1. The control group of healthy subjects and the patients underwent psychological and cognitive testing, and we registered the visual evoked potentials during the contrast enhancement of the chessboard. All the data were processed using discriminant analysis. Additionally, the amplitude–temporal characteristics of the VEPs and the data of the Cattell test were processed using factor analysis. Then, all the obtained results were divided based on the VEP amplitudes into augmentors and reducers.

## 3. Results

**Figure 2:**
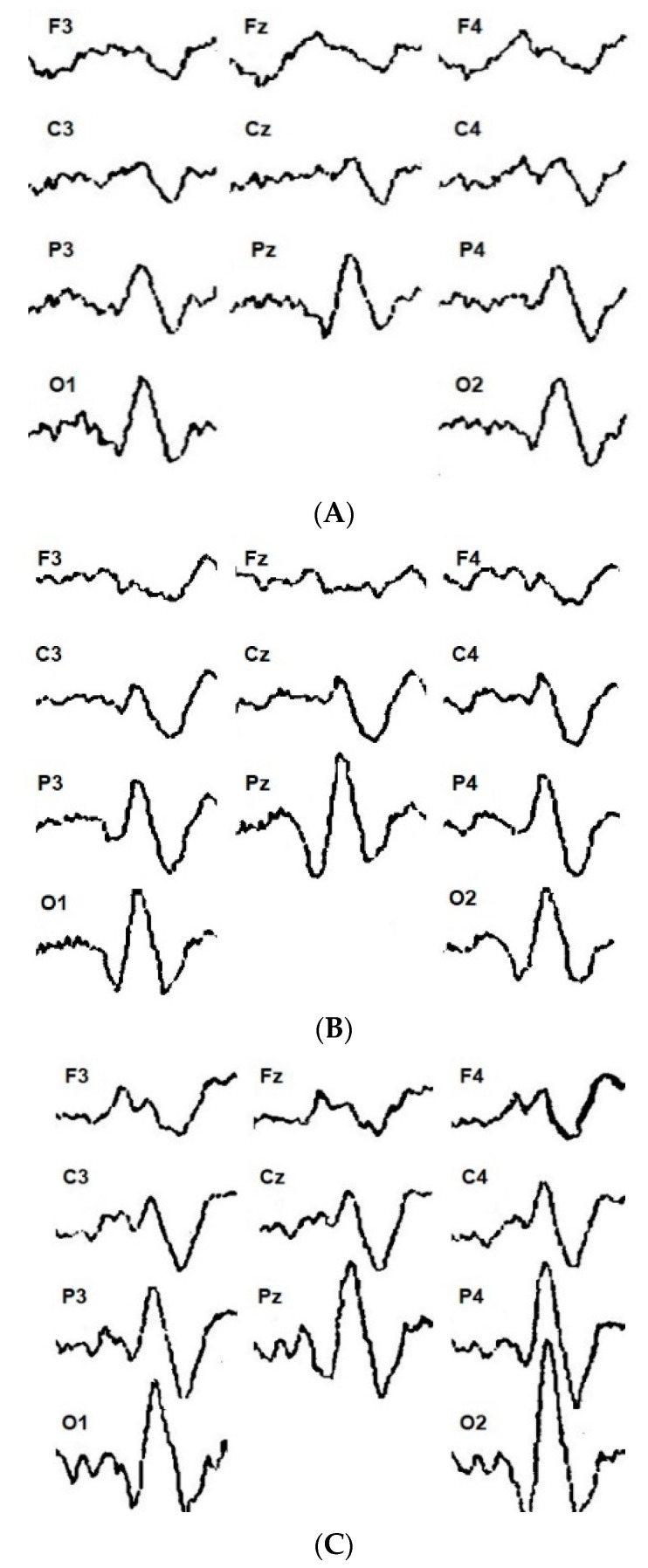
Distribution of visual evoked potential for augmentation during the presentation of reversible checkerboard patterns of minimum (**A**), average (**B**), and maximum (**C**) power across the sites of the 10/20 system (O_2_, O_1_, P_4_……F_3_).

**Figure 3:**
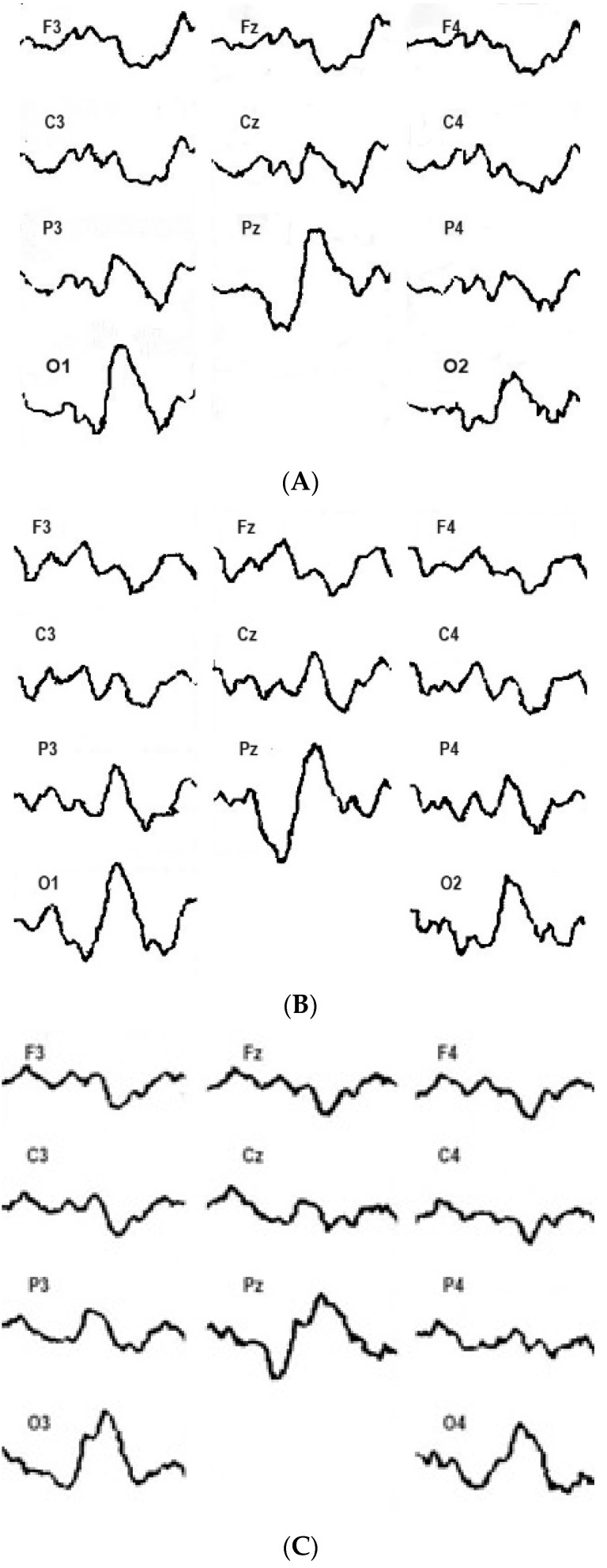
Distribution of visual evoked potential for reducing during the presentation of reversible checkerboard patterns of minimum (**A**), average (**B**), and maximum (**C**) power across the sites of the 10/20 system (O_2_, O_1_, P_4_……F_3_).

Depending on the type of visual evoked potential response to the increase in checkerboard contrast power, all the subjects in the control and study groups were divided into groups. The first group (21 patients and 29 healthy subjects) showed a constant increase in VEP amplitude with an increase in the checkerboard contrast power (Figure 2). These subjects were called “augmenters” (A) in accordance with the previously proposed classification. The second group (18 patients and 23 healthy subjects) initially demonstrated an increase in the VEP amplitude, but with a subsequent increase in the contrast of the checkerboard patterns, the amplitude of the evoked responses was reduced (Figure 3). Accordingly, these participants were called “reducers” (R).

**Figure 4:**
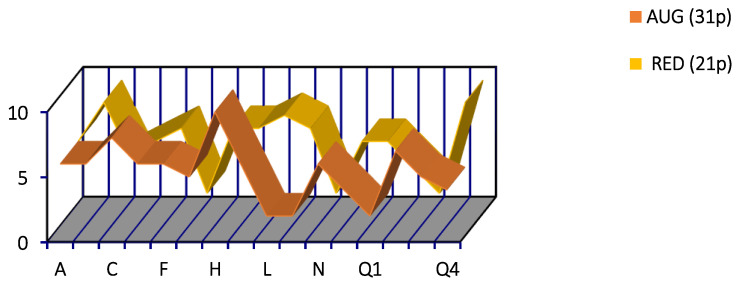
Factor characteristics of healthy subjects using the Cattell test. Group A—31 persons, R—21 persons. On the ordinate scale—points, abscissa—factors C (emotional instability/stability) and Q4 (tension) are the factors being assessed.

The assessment of factors C (emotional instability/stability) and Q4 (tension) characterizes the individuals being examined as follows (Figure 4). The importance of the behavioral characteristics associated with relaxation and tension was characterized on a scale of 1–6 points. At the same time, the behavioral indicators of emotional stability were given a point within the range of 5–9. In order to establish the relationship between the emotionally driven behavioral characteristics and the amplitude of the visual evoked responses of the brain, the subjects of the control group were divided into two groups. Thirty-one subjects were in the group with high values for tension (4–6 points), while twenty-one subjects were added to a group with low scores for this indicator (1–3 points).

A psychological experiment may assume the presence of an intermediate (relatively neutral) group. However, in order to clarify the patterns of response mechanisms to multi-contrast afferentation, it is appropriate to only create two opposing groups. It should be noted that higher values (4–6) for factor Q4 corresponded to lower values for factor C (5–6). By contrast, low tension scores (1–3) corresponded to high emotional stability scores (7–9).

Depending on the participants’ emotional state, there were certain VEP patterns in response to the increased contrast of the chessboard. Fundamentally, the following results were obtained: On the one hand, contrast enhancement was directly proportional to VEP amplitude; this relationship disappeared over time. On the other hand, the opposite was also true. When the contrast of pattern afferentation increased, it was inversely proportional to the VEP responses. The typical VEP dynamics are presented in Figure 2 and Figure 3.

**Figure 5:**
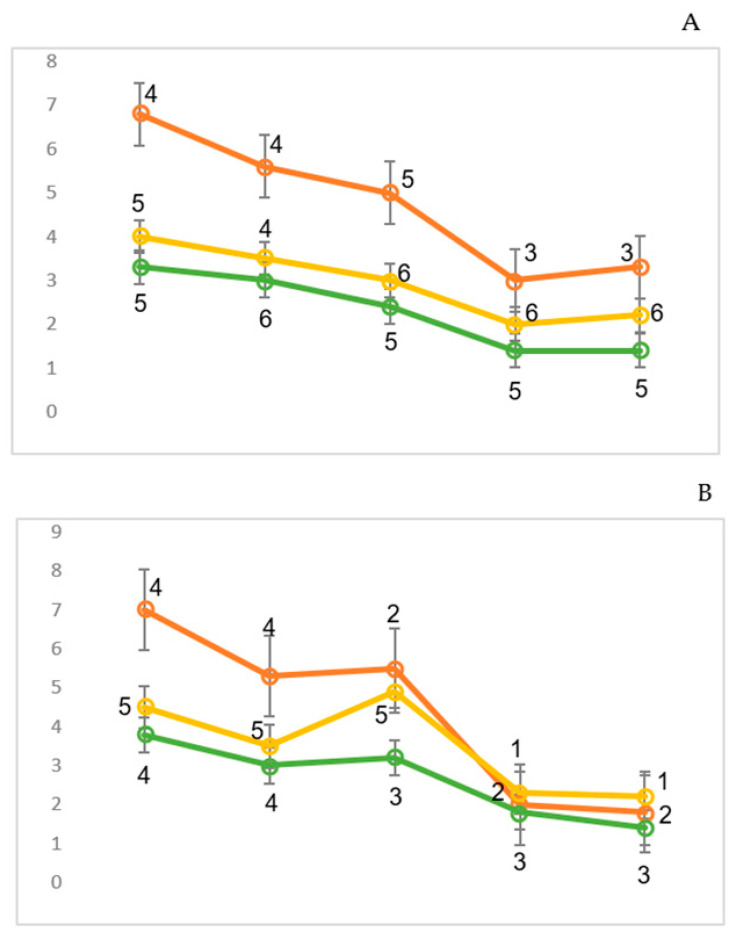
Characteristics of the amplitude factors of the visual EP component N_70_ with minimum (green), average (yellow) and maximum (red) contrast of chessboard squares. (**A**)—augmentation, (**B**)—reduction. Amplitude, μV—ordinate; registration points according to the 10/20 system (O_2_, O_1_, Pz, F_2_, F_1_)—abscissa. The numbers above the lines reflect the ordinal numbers of the factors.

**Figure 6:**
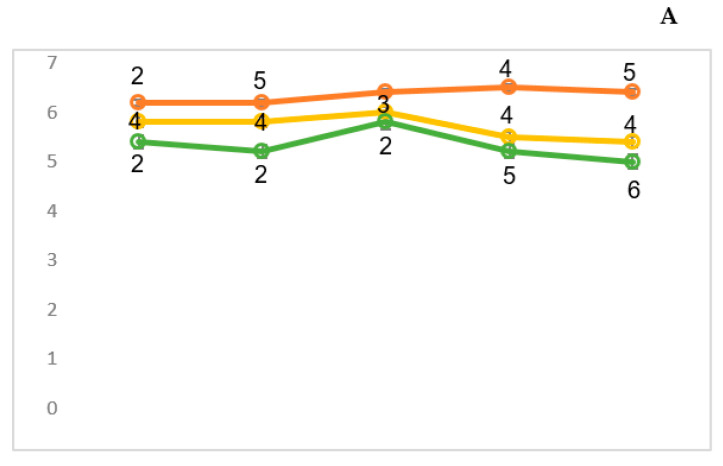

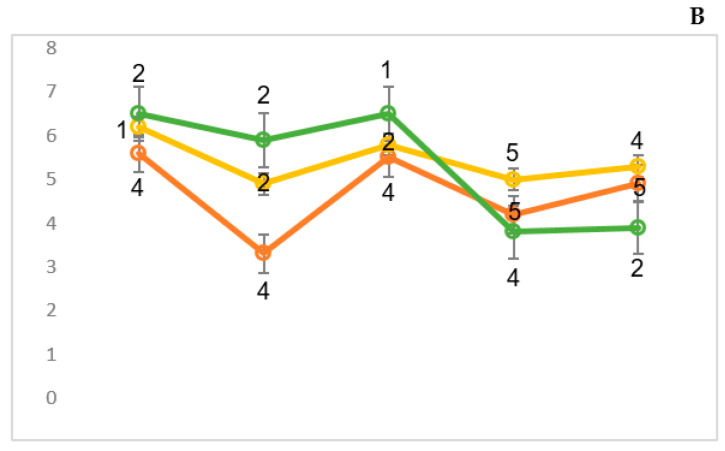
Characteristics of the amplitude factors of the visual EP component N_150_ with minimum (green), average (yellow) and maximum (red) contrast of chessboard squares. See Figure 5. (**A**)—augmentation, (**B**)—reduction. Amplitude, μV—ordinate; registration points according to the 10/20 system (O_2_, O_1_, Pz, F_2_, F_1_)—abscissa. The numbers above the lines reflect the ordinal numbers of the factors.

The visual EP amplitudes of the studied groups featured both N_70_ (Figure 5) and N_150_ waves (Figure 6). As a general tendency, it can be stated that for all the subjects, the spatial characteristics of the N_70_ wave differed slightly from each other. At the same time, the minimum amplitudes were recorded when presenting low-contrast chessboard squares. An increase in power for all the subjects was accompanied by an increase in the amplitude of the N_70_ wave. In addition, the spatial dynamics of the N_70_ wave were similar in both groups. The maximum amplitude was recorded in the posterior brain areas, predominantly on the right. Additionally, high values for the factor analysis of amplitudes were observed in the right parietal–occipital region, indicating minimal dispersion. The amplitude factors were also more dispersed in the frontal registration regions, especially for the subjects with low scores for the Q4 factor (low factor numbers). In addition, in the frontal areas of the subjects in this group, a higher increase in N_70_ amplitude was recorded when the subjects were exposed to moderate-contrast chessboard squares compared to the maximum-contrast ones (Figure 5).

The spatial characteristics of the N_150_ wave showed more interesting differences between the studied groups. Thus, if there was a direct relationship between the contrast of the chessboard cells and the N150 amplitude (Figure 5) for the subjects with high Q4 values (“tension and relaxation” (Figure 4)), then for the other subjects with low Q4 values, amplitude inversion was recorded (Figure 6). This group was also characterized by a minimal N_150_ amplitude in the posterior brain areas when presenting the most contrasting patterns. At the same time, in these areas of the brain, the minimal-contrast stimulus contributed to the formation of the most pronounced N_150_ components, with a noticeable predominance of amplitude in the frontal region on the right. The N_150_ wave value was intermediate in response to the average-contrast chessboard patterns. Additionally, in the anterior half of the brain, the amplitude of this wave was at its maximum. Generally, in all the study groups, the N_150_ component values in the posterior regions of the cerebral cortex exceeded the amplitude values in the frontal cortex, as also evidenced by the higher scores for the factors (Figure 5 and Figure 6).

**Figure 7:**
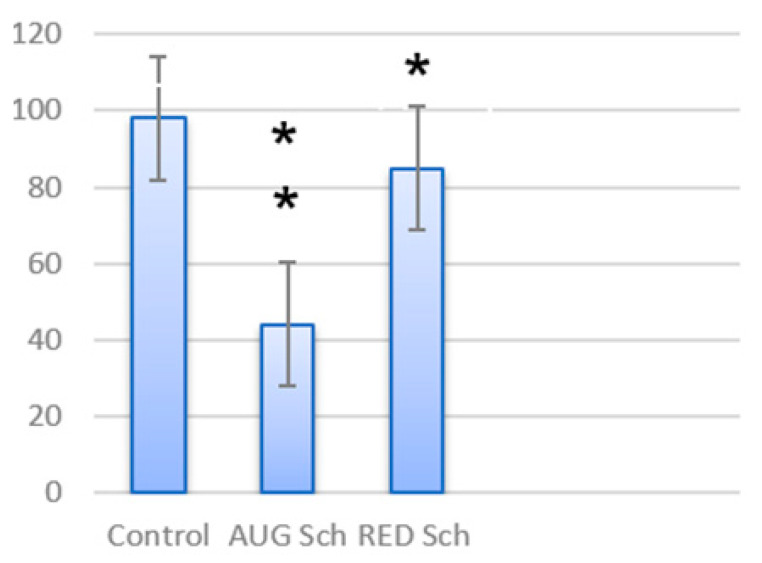
Results of correct and incorrect perception (%) of Perret figures. *—*p* < 0.05, **—*p* < 0.01 (compared with control group). Note. AUG—“augmentors” group, RED—“reducers” group.

**Figure 8:**
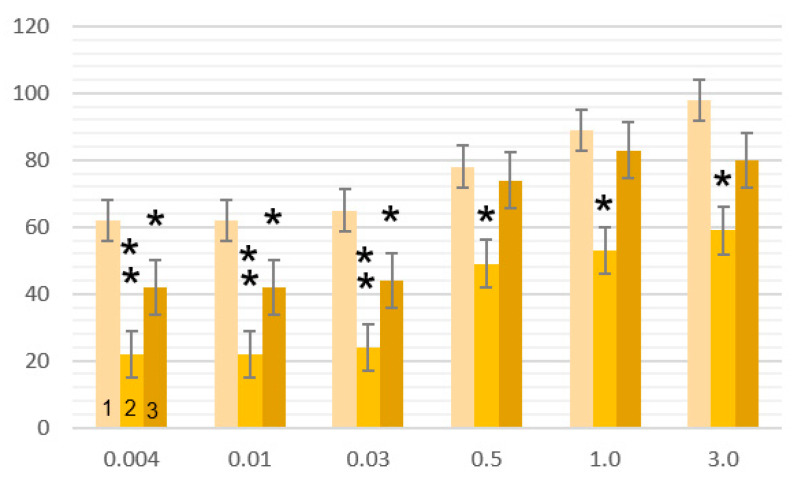
Recognition values (ordinate, %) of images of figures with the absence of some features under conditions of perception-time deficit: 0.004, 0.01….3.0 s (abscissa). Note. *—*p* < 0.05, **—*p* < 0.01 (compared with the control group). Histograms: 1—control, 2—group A (“augmenters”), 3—group R (“reducers”).

The visual–cognitive testing results obtained under time pressure were compared across all subjects. These were formed by spatially analyzing each component’s visual evoked potential amplitudes when presenting increasingly contrasted chessboards. In general, regardless of the nature of the VEP response and the modality of the visual objects, the healthy subjects were better at image perception (Figure 7 and Figure 8). In psychopathology, many differences associated with VEP responses have been recorded. Thus, in group R, the perception of the successive parts of the Perret figures approached the norm, while in group A, the number of correct responses was significantly lower (Figure 6; *p* < 0.01). Similar trends between the studied groups were noted when recognizing figures with missing elements, especially under time pressure (Figure 8). Moreover, the largest differences appeared when the test images were only shown for 0.004, 0.01, and 0.03 sec. Group R performed better than the healthy subjects did. When the test time was extended to 0.5, 1.0, and 3.0, the responses of group R approached those of the healthy subjects, while in group A, significant differences remained.

## 4. Discussion

The results of this study are discussed in accordance with existing concepts and theories related to emotional–motivational activation, inhibition mechanisms in the central nervous system, changes in sensitivity during behavioral stimulation, and the testing of executive cognitive functions under difficult conditions [2,33,38,39]. The efficiency of the executive cognitive functions providing sensory perception is associated with the generalized functioning of information processes in the central nervous system, which is also reflected in the dynamics of sensitivity when analyzing structural images [37,38]. The generalized response of the anterior cerebral cortex when presented with an average-contrast image of a chessboard may be associated with the higher rate of correct perception in the second group of patients with a psychopathology. This may be due to more synchronous functioning in the neural centers of the brain when processing important information. The switching mechanism causing a reduction in VEP amplitude is triggered by the response to the more intense information stimulus, which may indicate the stimulus’ biological insignificance. By contrast, a slower switching mechanism reflects the inhibitory processes of the brain, which affects the implementation of the executive cognitive functions of visual perception.

A mechanism in the study of sensory perception with extreme signal amplitudes is the coordination of nerve centers in the regulation of the excitation/inhibition ratio in the central nervous system [5,15,40]. In turn, coordination activity is interconnected with the basic law of psychophysics, where the amplitude of the response is determined by threshold sensitivity. A high threshold of absolute sensitivity results in a better response to more intense stimulation. By contrast, a highly sensitive system with a low absolute sensory threshold launches a “program” that protects against “overload”. Despite an increase in stimulus intensity, poor responses have been evoked [4,12].

In many studies on the A/R mechanism, unimodal signals of variable intensity (amplitude) have been used as stimuli, where only the physical characteristics of the stimulus were gradually changed [5,12,33,35]. The use of reversible chessboard patterns as a stimulator has some fundamental features. The impact on the visual system is considered to be low compared to that caused by the potential [3,4].

In an oddball paradigm study with checkerboard patterns, during ERP recording, the authors assessed subjects’ positive and negative emotions to still images [20]. The participants were presented with two types of stimuli: a frequently targeted stimulus with a black-and-white image of a chessboard was presented in 76% of cases, and a rarely targeted oddball stimulus with an image of traffic was presented in 24% of cases. The ERP responses were recorded [20]. Consistent with RST, it was hypothesized that individuals with stronger versus weaker BASs would have larger mean N_100_ and N_200_ amplitudes, reflecting greater early and late attention to positive images. Because the FFFS system is sensitive to punishment, those with a stronger FFFS may elicit larger mean N_100_ and N_200_ amplitudes when presented with negative images [17,34,39].

The authors associate the change in sensitivity regardless of the level of sensory signal processing with the findings from these different studies. Thus, the above-described mechanism of switching (reduction) during the analysis of increasingly intense information, which forms the basis of the A/R phenomenon, is sometimes associated with biochemical changes. In particular, the concentration of substances involved in stress reactions, as well as endorphins such as serotonin in pleasure reactions, may be important [1,41]. The individual behavioral characteristics of subjects and even complex animals are increasingly being associated with the above-mentioned biochemical indicators [4,32,37]. The mechanisms of stress and pleasure are integrated with emotion, which is realized through reticulo–thalamo–frontal interaction, with the obligatory participation of the hypothalamus centers. If the hypothalamus provides a hormonal effect on integration processes, then the anterior regions of the brain act as the information component of emotions [5].

If the results of behavioral and neurophysiological studies are relevant for a relatively long period of time, we should compare the trends between bioelectrical activity (EEG and EPs) and behavior. We should record biosignals in most informative frontal channels to compare behavioral and cognitive information [9,12,14]. In particular, factor Q4 (Cattell’s questionnaire) has a direct connection with the N_70_ and N_150_ components of visual evoked potentials. In addition, results have shown that the activation of the switching mechanism with reduced stimulation is more commonly manifested in extroverts [3,4]. This study showed that the initial reactions of all the subjects to more contrasted chessboard cells were similar and consisted of an increase in the amplitude of the N_1_/P_1_ VEP complex. For group A, this mainly correlates to the posterior regions of the brain (Figure 2); however, in group R, the increase in VEP amplitude was more pronounced in the parietal and central regions of the brain (Figure 3). This relationship allowed us to conclude that the initial increase in power had sufficient informational significance for all the subjects. In only one group of subjects did this manifest mainly in the projection zone of the visual system, while in the other group, the responses were predominantly in the associative cortex.

The visual evoked potential reactions to the increase in stimulus intensity are represented by a generalized increase and decrease in amplitude in groups A and R, respectively; this finding is reminiscent of the previously proposed concept of “switching points” [1,2]. In this theory, the central nervous system functions in search mode, continuously monitoring the absolute sensitivity of sensory perception when stimulators approach the switching points. According to the law of force and the variant pessimal protective inhibition [4,7,8,10], the switching mechanism is considered a process of transmission, where in some cases, the response corresponds to the law of force, and in others, an adaptive–protective mechanism is launched.

One explanation for the differentiation of evoked potential responses with increasing stimulus intensity is cortical prepulse inhibition (PPI), followed by the prepulse inhibition of perceived stimulus intensity (PPIPSI). Both these phenomena have been described in studies of visual and auditory sensory perception in clinical and non-clinical settings. PPIPSI is partly related to the allocation of attentional resources for monitoring the cortical recording channels that process stimulus intensity features, such as the N1–P2 complex of auditory evoked potentials [22,27,29].

To study the A/R mechanism in the perception of structural information, e.g., chessboard patterns, we must establish relationships between the neurophysiological and cognitive process data. Firstly, this study showed that the patients with a psychopathology had worse identification abilities; secondly, the second group of patients (R) was better at identification than the first group (A), regardless of the modality of visual images used (Figure 4 and Figure 5). These results confirm the nonspecific nature of cognitive executive functions in patients with schizophrenia detected under conditions that complicated identification. In addition, it was possible to compare the neurophysiological and behavioral signs of A/R and thus to assess the specific disorders of sensory identification mechanisms.

The perception of Perret figure fragments triggers three successive processes: the identification of the individual features of non-verbalized figures, the storage of a set of features of each fragment in short-term memory, and finally, 2D synthesis with subsequent recognition. For group R, the number of correct answers insignificantly differs from those of the group of healthy subjects (*p* > 0.05), while group A is characterized by a significantly smaller number of correct answers (Figure 6; *p* < 0.05). A similar but more pronounced tendency is observed in the recognition of images with the absence of some features (Figure 7), which involves turning on the invariant mechanism of image recognition.

The productivity of the leading mechanisms of sensory identification—invariant signal assessment, short-term visual memory, spatial analysis, and synthesis—is associated with the peculiarities of perceiving increasingly complex structural information. In particular, a more pronounced reaction to the stimulus of medium intensity in the anterior frontal cortex is associated with higher rates of the recognition of visual images in the group of patients with schizophrenia (R), indicating the synchronization of the cerebral hemispheres when processing significant information. The adaptive switching mechanism, which is activated by an increase in intensity, manifests as a reduction in the EP amplitude, indicating that this information stimulus is less biologically significant. On the contrary, the absence of such a mechanism indicates inert (inhibitory) brain processes, which are accompanied by an increase in the threshold of the sensory identification of images.

Regarding the localization of the adaptive switching mechanism in the brain, activity has been noted in the posterior sections, i.e., the parietal and occipital regions of the brain. Previously described data in favor of interhemispheric asymmetry with an increase in amplitude in the right occipital lobe are associated with the results of other studies when recording VEPs in response to reversible chessboard patterns both in typical [13] and pathological groups [20].

Other authors also assessed the impact of individual differences in the BAS and the FFFS on the attention of young female drivers to positive and negative anti-speeding television advertising images [20]. It was found that reactivity to rewards and the BAS were accompanied by significantly larger N_200_ ERP amplitudes in the vertex (Cz), which suggests the strong automatic orientation of attention when presented with negative images. However, while the BAS had no significant effect on positive reactions to images, differences in the EEG alpha range were established [33]. It is also possible that women score higher on certain subscales of the BAS, such as reward reactivity, compared to men, and this result is consistent with other studies [13].

In this study, according to Cattell’s factors, there is an emotional connection between the intensity of the presented chessboard cells as an information stimulus and the amplitude of the N_150_ visual EPs (Figure 5 and Figure 6). The initial responses to the increase in power in all the subjects were similar, with a generalized increase in the N_150_ VEP amplitude in groups A and R in the anterior areas of the brain. We conclude that the initial increase in the intensity of the information stimulus was sufficiently important for all the subjects. Further, the responses to an increase in intensity—presented in the form of consistent, generalized increases and decreases in N_150_ VEP amplitude in the subjects under emotional stress and the individuals who are not under emotional stress, respectively—are similar to a number of previously proposed mechanisms regulating structural afferentation. Thus, the results of this study indicate the presence of a switching mechanism in response to increasingly intense structural stimuli, which is associated with the subject’s emotional state and is provided mainly by the frontal information neocortex. Due to the ambiguous results and the lack of large comparative studies in this area, future research is needed to clarify the potential influence of sensory sensitivity on rewards, punishment, attention, and executive cognitive functions.

In addition, some mechanisms responsible for varied cognitive functions in subjects with different EP responses to increased stimulation are related to cortical prepulse inhibition [22,24,28]. On the one hand, this inhibition reduces the EP amplitude with increased stimulation, and on the other hand, it triggers reverse corticofugal inhibition, which tunes the selective attention system. This may explain the occurrence of higher executive functions with EP reduction, especially in psychopathology.

Future studies related to changes in the sensitivity of sensory systems and cognitive functions with increasingly intense sensory stimulation should be conducted, with several possible research directions. Firstly, on the psychophysiological side, research in psychoneurological clinics should be performed to address diagnostic and even expert and prognostic concerns. Secondly, healthy subjects should be studied in the field of professional psychological selection. Thirdly, technical systems for preimpulsation should be developed to adjust the cognitive functions observed by specialist operators.

## 5. Conclusions

An increase in the intensity of a contrasting stimulus resulted in two spatial distributions of visual evoked potentials. In the first case, a direct relationship between the intensity of the stimulus and the amplitude of the N_70_ and N_150_ VEP waves was recorded (group A); in the second case, the direct relationship between the stimulus and the amplitude of the VEPs was reversed (group R).

The classification of VEP responses has a clear relationship to the personal and cognitive characteristics of performance. For the healthy subjects, with low scores for factor Q4 (tension/relaxation), and conversely high scores for factor C (emotional stability/instability) in Cattell’s questionnaire, a reversible relationship between the amplitudes of the stimulus and the N_150_ VEP component was recorded in the occipital–parietal regions of the brain. In the frontal areas, a switching regulatory mechanism was noted, which caused a decrease in the N_150_ VEP amplitude.

For healthy subjects with high levels of stress (factor Q4) and low scores for emotional stability (factor C), only a direct relationship was recorded between the increase in the amplitudes of the stimulus and the N_70_ and N_150_ VEP components.

The capacity for visual recognition, regardless of personality and neurophysiological classification, was higher in the healthy subjects. A number of differences were recorded among the patients with a psychopathology. In group R, the indicators of short-term visual memory and the formation of Gestalt images approached the control values (*p* > 0.05), while in group A, the number of correct answers was significantly lower (*p* > 0.05). The same trend was noted for the patients with psychopathologies in the invariant recognition of visual images.

## Data Availability

Data available on request due to restrictions of the privacy and ethical.
